# Purification and characterization of an extracellular β-xylosidase from *Pseudozyma hubeiensis* NCIM 3574 (PhXyl), an unexplored yeast

**DOI:** 10.1186/s13568-016-0243-7

**Published:** 2016-09-15

**Authors:** Nutan Mhetras, Susan Liddell, Digambar Gokhale

**Affiliations:** 1 NCIM Resource Center, CSIR-National Chemical Laboratory, Pune, Maharashtra 411008 India; 2 University of Nottingham, Nottingham, LE12 5RD UK

**Keywords:** Unexplored yeast, *Pseudozyma hubeiensis*, β-Xylosidase, Metal and ethanol tolerant enzyme

## Abstract

This paper reports on the production of β-xylosidase from an unexplored yeast, *Pseudozyma hubeinsis*. The expression of this enzyme could be induced by beech wood xylan when the yeast was grown at 27 °C. The enzyme was purified to homogeneity as a glycoprotein with 23 % glycosylation. The purification protocol involved ammonium sulphate precipitation, QAE-Sephadex A50 ion exchange chromatography and sephacryl-200 column chromatography which resulted in 8.3-fold purification with 53.12 % final recovery. The purified enzyme showed prominent single band on SDS-PAGE. It is a monomeric protein of 110 kDa molecular weight confirmed by SDS-PAGE followed by MALDI-TOF mass spectrometry (112.3 kDa). The enzyme was optimally active at 60 °C and pH 4.5 and stable at pH range (4–9) and at 50 °C for 4 h. Chemical modification studies revealed that active site of the purified enzyme comprised of carboxyl, tyrosine and tryptophan residues. The carboxyl residue is involved in catalysis and tryptophan residue is solely involved in substrate binding. The best match from the search of the NCBInr database was with gi|808364558 glycoside hydrolase of *Pseudozyma hubeiensis* SY62 with 26 % sequence coverage confirming that it is a glycoside hydrolase/beta-glucosidase. From the search of customized SWISSPROT database, it was revealed that SWISSPROT does not contain any entries that are similar to the purified enzyme.

**Electronic supplementary material:**

The online version of this article (doi:10.1186/s13568-016-0243-7) contains supplementary material, which is available to authorized users.

## Introduction

Species of *Pseudozyma* belong to Ustilaginales as suggested by morphological (Boekhout 1987) and molecular studies (Begerow and Bauer 2000; Fell et al. 2000). Assimilation of inositol was one of the diagnostic phenotypic criteria for the genus *Pseudozyma*. However, *Pseudozyma hubeinsis* does not assimilate inositol and hence it differs from all other species of the genus reported so far. Therefore, Wang et al. (2006) suggested the emendation of the diagnosis of *Pseudozyma* which recommends the deletion of the positive inositol assimilation reaction from the diagnostic of this genus. We were the first to isolate this yeast from decaying sandal wood (Bastawde et al. 1994) but it was identified in 2008 by National Collection of Yeast Cultures (NCYC) as *P. hubeinsis* using 26 rDNA D1/D2 sequencing and standard taxonomic tests. It was deposited at NCIM Resource Center, National Chemical laboratory, Pune with an accession number NCIM 3574. Wang et al. (2006) isolated *P. hubeinsis* and *P. shanxiensis* from wilting leaves of various plants in China.

*Pseudozyma hubeinsis* remained unexplored till today in relation to hydrolytic enzymes production. We were the first to report on the cellulase free xylanase production by this yeast strain (Bastawde et al. 1994) followed its application to hydrolyse xylan from various agro-waste materials to produce xylose which can be driven further to value added chemicals (Gokhale et al. 1998). The xylanase was also used to remove the hemicellulosic fractions from bleached or unbleached pulp, as well as the jute fibres used in textile industries without disturbing the cellulose micro-fibril structures. Two distinct xylanases (PhX20 and PhX33) from *P. hubeinsis* NCIM 3574 were purified to homogeneity having molecular masses of 20.1 and 33.3 kDa respectively (Adsul et al. 2009).

Plant biomass is abundantly available renewable resource which is composed of mainly cellulose and hemicellulose. These carbohydrate polymers can be converted to their respective monomers that will be further converted to second generation bioethanol. The second generation bioethanol has received great attention since it is derived from non-food based sources. Many strategies have been introduced into industrial processes to produce second generation bioethanol with economically viable process. The main strategy is the physicochemical pre-treatment to disrupt the lignocellulosic structure to enhance the cellulose and hemicellulose accessibility. Using this strategy, industrial processes currently used to produce bioethanol consists of fermenting glucose, enzymatically released from cellulose, by *Saccharomyces cerevisiae*. However, hydrolysis of hemicellulose in biomass and xylose fermentation at industrial scale are also crucial to enhance the biomass conversion yields. The complete hydrolysis of xylan requires the coordinated action of endo-β-1,4-xylanases (EC 3.2.1.8) and β-xylosidases (EC 3.2.1.37). The endoxylanases cleave the xylan to produce soluble oligosaccharides which are further degraded to xylose by β-xylosidases. Most of the commercial enzymatic preparations are deficient in β-xylosidases (Bao et al. 2012).

Fungi and bacteria remain the attractive sources of robust industrial enzymes since they are recovered from fermentation broth to ease downstream processing. The studies on xylanolytic enzymes are prompted by the importance of hemicellulose as abundant carbohydrate in lignocellulosic biomass. This abundant natural carbohydrate is underutilized resource either as a renewable bioenergy source or source complex materials. Very few yeasts have been reported to possess complete xylanolytic enzyme systems which degrade xylan to xylose (Chevaz et al. 2006). The β-xylosidase is a rate limiting enzyme since it acts on xylobiose which is inhibitor of endoxylanase. In addition to saccharification, these enzymes play role in production of ethanol from pentoses, xylitol and polyalcohols which find application as natural food sweeteners, dental caries reducers and sugar substitutes in diabetes (Saha 2003). In addition, these glycosidases including β-xylosidases play a major role in improving wine aroma complexity (Padilla et al. 2016). Our earlier reports suggest that *P. hubeinsis* possesses complete xylanolytic enzyme system (Bastawde et al. 1994; Adsul et al. 2009). This paper reports the production, purification and characterization of extracellular β-xylosidase from *P. hubeinsis* NCIM 3574 (PhXyl), an unexplored yeast strain.

## Materials and methods

### Chemicals

The *p*-nitrophenyl-β-d-xylopyranoside (*p*NPX), *p*-nitrophenyl-β-d-glucopyranoside (*p*NPG), beech wood xylan, N-ethylmaleimide (NEM), iodoacetate, phenyl methylsulfonyl fluoride (PMSF), diethyl-pyrocarbonate (DEPC), 1 ethyl-3-(3 dimethyl aminopropyl) carbidiimide (EDAC), 2-4-6 trinitrobenzenesulfonic acid (TNBS), 5-bromosuccinimide (NBS), N-acetylimidazole (NAI), citraconic anhydride, acetic anhydride, phenyl glyoxal, HEPES and MES, QAE-Sephadex A-50, Sephacryl-200 Coomassie Brillient Blue G-250, Bromophenol Blue were obtained from Sigma-Aldrich, St. Louis, USA. SDS-PAGE markers were purchased obtained from Invitrogen. All other chemicals were commercially sourced and used without further purification.

### Microbial strain, growth media and enzyme production

*Pseudozyma hubeinsis* NCIM 3574 was obtained from NCIM Resource Center, CSIR-National Chemical Laboratory, Pune, India. It is also deposited in NCYC, UK with an accession number NCYC 3431. The strain was maintained on MGYP agar medium consisting of 0.3 % malt extract, 1 % glucose, 0.3 % yeast extract, 0.5 % peptone and 2 % agar and it was sub-cultured once in every 15 days. The fermentation medium used for enzyme production consisted of 0.05 % NaNO_3_, 0.05 % KCl, 0.05 % MgSO_4_, 0.02 % K_2_HPO_4_, 0.1 % yeast extract, 0.5 % bacto-peptone and 2 % xylan. The initial pH of the medium was adjusted to 5.5 prior to sterilization. For enzyme production, the submerged fermentation (SmF) was carried out in 250-mL Erlenmeyer flasks with 70 mL of the fermentation medium. The flasks were inoculated with 5 % inoculum prepared in MGYP liquid medium and incubated at 27 °C with shaking at 170 rpm. The cell growth was harvested after 120 h by centrifugation (7000×*g*, 15 min) and the supernatant was used as a source of crude enzyme. To see the effect of temperature on enzyme production, the SmF was carried out at different temperatures (25–30 °C) and samples were removed at different time intervals, centrifuged and analyzed for enzyme activity.

### Analytical methods

β-Xylosidase (β-d-xylan xylohydrolase, EC 3.2.1.37) activity was estimated using *p*NPX as substrate in 50 mM citrate buffer, pH 4.5. The total 1 mL of reaction mixture consisted of 0.9 mL of *p*NPX (0.5 mg mL^−1^) and 0.1 mL of suitably diluted enzyme. The reaction was initiated by the addition of enzyme followed by incubation at 60 °C for 30 min. The reaction was terminated by the addition of 2 mL of 2 % sodium carbonate and the liberated *p*-nitro phenol was measured at 410 nm. One unit of enzyme activity was defined as the amount of enzyme required to liberate 1 µmol of *p*-nitro phenol from the substrate. Protein was determined according to Lowry method (Lowry et al. 1951) with bovine serum albumin as standard. Glycoprotein content of the purified enzyme was determined by the phenol–sulfuric acid method (Dubois et al. 1956) with d-mannose as the standard.

### Native polyacrylamide gel electrophoresis and zymogram of β-xylosidase

For zymogram staining, the crude enzyme preparation was fractionated by native polyacrylamide gel electrophoresis (PAGE) using 10 % acrylamide as resolving gel and 4 % stacking gel (Laemmli 1970). The β-xylosidase activity in the gel was detected by developing zymogram against 10 mM 4-methylumbelliferyl-β-d-xyloside as substrate prepared in 50 mM sodium citrate buffer (pH 4.5). Upon completion of electrophoresis, the gel was immersed in substrate solution for 45 min at 50 °C in the dark. The β-xylosidase bands in the gel were visualized under UV light using Gel Documentation system (Syngene).

### Purification of β-xylosidase

The fermented broth was centrifuged at 7000×*g* for 10 min and the supernatant was concentrated by ammonium sulfate precipitation at 90 % saturation at 4 °C with constant stirring and left overnight. The concentrated crude extract (5 mL) was loaded onto a QAE-Sephadex A-50 column (30 × 2.5 cm) pre-equilibrated with 20 mM glycine–NaOH buffer (pH 8.0). The column was then washed with the same buffer to confirm that the flow-through fractions showed no activity. The bound proteins were then eluted with 0.3 M NaCl at a flow rate of 1 mL min^−1^ and the fractions (3.0 mL) with β-xylosidase activity were pooled and then concentrated. The concentrated fraction was dialyzed extensively against the 10 mM glycine–NaOH buffer (pH 8.0). The dialyzed fraction was freeze dried and dissolved in minimal volume of 10 mM glycine–NaOH buffer (pH 9.0). This concentrated fraction was applied to Sephacryl S-200 column (1.5–110 cm) previously equilibrated with 10 mM glycine–NaOH buffer (pH 8.0) and the fractions were collected at a flow rate of 0.2 mL min^−1^. Fractions (1.8 mL) showing β-xylosidase activity were pooled together, concentrated by freeze drying and the purified concentrated enzyme was stored at 20 °C till further use.

### Enzyme characterization

The molecular mass of PhXyl was determined by 10 % SDS–PAGE. The molecular mass of the native enzyme was determined by matrix assisted laser desorption ionisation time-of-flight (MALDI-TOF) mass spectrophotometry, using Voyager DE-STR (Applied Biosystems, USA) equipped with a 337 nm nitrogen laser. The matrix was prepared in deionized water containing sinapinic acid (10 mg mL^−1^), 50 % acetonitrile and 0.1 % TFA. The β-Xylosidase was mixed with matrix (1:1) and 2 µL of the sample was spotted on plate, dried at room temperature.

The optimum pH of the enzyme was determined by estimating enzyme activities at 65 °C in 50 mM citrate phosphate buffer at different pH values (2.5–6.0). The pH stability studies were performed by incubating the enzyme in 50 mM buffer systems with different pH values ranging from 2.0 to 9.0 (KCl–HCl buffer, pH 2.0; citrate phosphate buffer, pH 2.5–6.0; phosphate buffer, pH 7.0; glycine NaOH buffer, pH 8.0–11.0) at 30 °C. The residual enzyme activity was then assayed under standard assay conditions. The optimal temperature of the enzyme was determined by performing the enzyme assays at various temperatures (40–80 °C) in 50 mM citrate buffer (pH 4.5). Temperature stability of the enzyme was determined by pre-incubating the enzyme in 50 mM citrate buffer (pH 4.5) for 4 h at different temperatures (50–70 °C) followed by measuring the residual activity under standard assay conditions. The effect of heavy metals and EDTA on enzyme activity was determined by performing enzyme assays in presence of respective metal salts and EDTA at varying concentrations (0.1, 1.0 and 10 mM).

Substrate specificity studies were carried out using *p*NP-β-glucopyranoside and *p*NP-α-l-arabinopyranoside, *p*NP-β-xylopyranoside as substrates. The *K*_m_ and *V*_max_ values of purified PhXyl were determined under standard assay conditions using 0.23–5.52 mM of *p*NPX as substrate. The constant values were calculated by fitting data to nonlinear regression using Michaelis–Menten equation.

To determine the effect of xylose on catalytic activity, assays were carried out in presence of various xylose concentrations (25–200 mM) using *p*NPX under standard assay conditions. To confirm the type of inhibition, kinetic constants (*K*_m_ and *V*_max_) were determined using different inhibitor concentrations (10, 15 and 20 mM) of xylose at varying *p*NPX concentrations (0.23–5.52 mM) under standard assay conditions. The effect of ethanol on enzyme activity was studied by incubating the enzyme in presence of ethanol at various concentrations (5–30 %, v/v) and the activity was determined at 40 and 60 °C under standard assay condition. The activity assayed in absence of ethanol was recorded as 100 %.

### Chemical modification studies using group specific reagents

Purified PhXyl (5 µg each) was incubated with various amino acid functional group specific reagents in 1 mL of the total reaction mixture. Chemical modification studies were performed under the conditions given in Table 5. After 30 min incubation at 30 °C, residual activity of enzyme samples was determined under standard assay conditions.

Modification of carboxyl residue was performed by incubating β-xylosidase (10 µg) with varying concentrations of EDAC (50–200 mM) in 1 mL of 50 mM MES/HEPES buffer (75:25), pH 6.0 at 30 °C. The control was kept without addition of EDAC. Samples were withdrawn after suitable time intervals and the reaction was terminated by addition of 1 mL of 50 mM citrate buffer, pH 4.5. The residual activity of modified enzyme was determined under standard assay conditions. Tryptophan residues were modified by incubating purified enzyme with increasing concentrations of NBS (0.1–1.0 mM) in 50 mM of sodium citrate buffer, pH 4.5 at room temperature. After 10 min, the aliquots were removed for analysis of residual enzyme activity. Tyrosine residue were modified by incubating purified enzyme with increasing concentration of N-acetyl-imidazole (10–50 mM) in 50 mM Sodium borate buffer, pH 7.6. Substrate protection studies were carried out by incubating the β-xylosidase with excess amount of substrate *p*NPX for 10 min followed by treatment with corresponding modified reagent. The residual enzyme activity was assayed under standard assay conditions.

### Mass spectrometric analysis of the purified protein

Proteins in gel bands were reduced, carboxyamidomethylated and digested with Trypsin Gold (Promega) on a robotic platform for protein digestion (MassPREP station, Waters). Resulting peptides were analysed by ESI–MS/MS after on-line separation on a C18 reversed phase, 75 μm inner diameter, 15 cm column (Jupiter 4 µm Proteo 90 Å, Penomenex, column made in-house, courtesy of David Tooth, UoN). Peptides were delivered via a CapLC HPLC attached to a Q-TOF2 mass spectrometer equipped with a nano-electrospray source (Waters) and operated with MassLynx Version 4.0 acquisition software. ProteinLynxGlobalSERVER software Version 2.1 (Waters) was used to generate a peak list file of un-interpreted fragment mass data which was used to search against all entries in the NCBInr (version 20151016) and SWISSPROT databases using the MASCOT search engine (http://www.matrixscience.com). Carbamidomethylation of cysteine and oxidation of methionine were set as variable modifications. One missed cleavage by trypsin was accepted. Only protein identifications with probability-based MOWSE scores above a threshold of p < 0.05 were accepted.

## Results

### Production of PhXyl and its purification

**Fig. 1:**
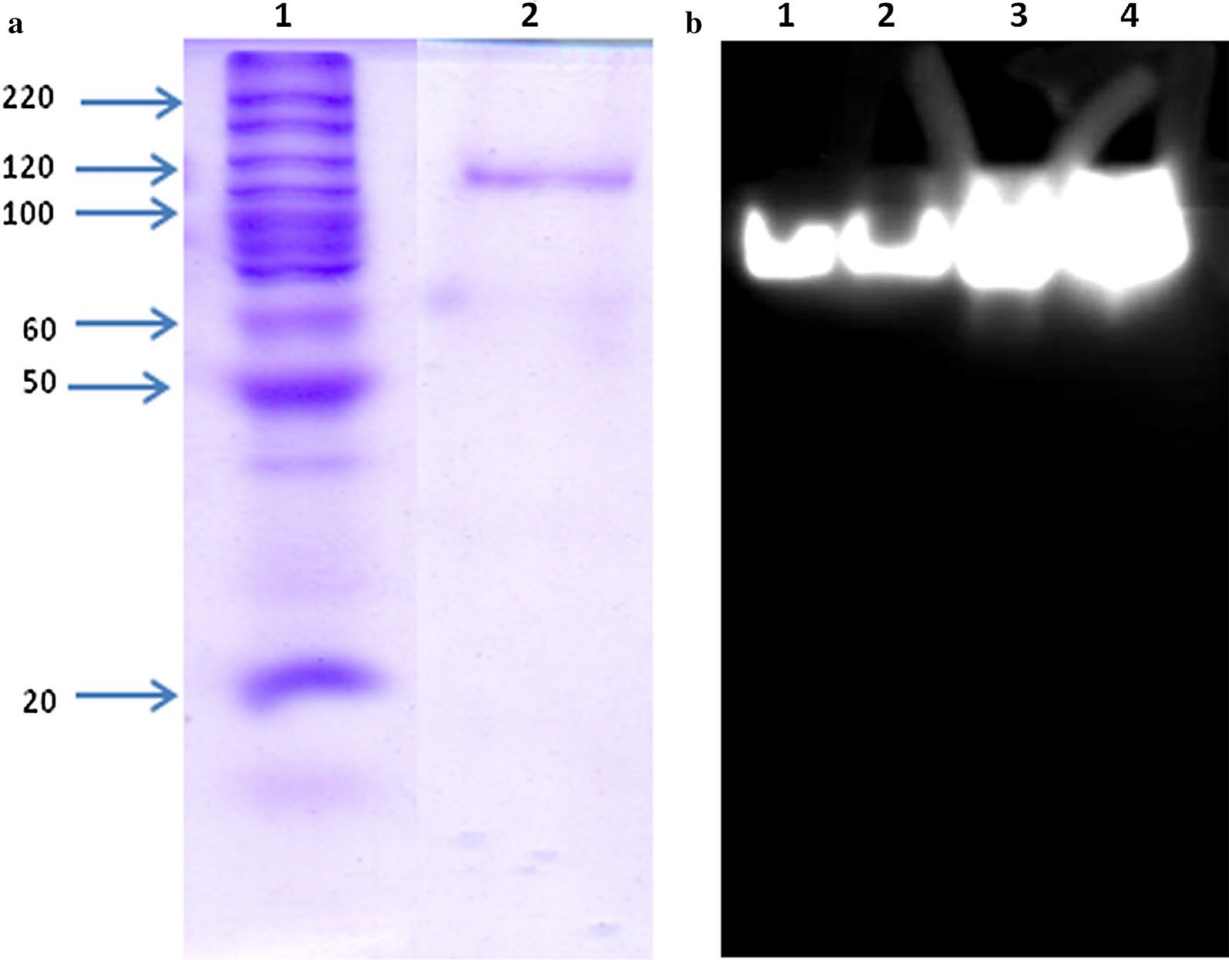
**a** SDS PAGE of the purified PhXyl. *Lane 1* Molecular weight standard, *lane 2* purified β-xylosidase. **b** Zymogram staining of PhXyl. *Lane 1* and *2* 10 µg of crude enzyme, *lane 3* and *4* 25 µg of crude enzyme

Our earlier report demonstrated that *P. hubeinsis* NCIM 3574 produces extremely less amount β-xylosidase when grown on xylan containing media at 30 °C. We also found that it grows significantly even at low temperatures and hence we evaluated β-xylosidase production at lower temperatures (25–30 °C) to know whether it produces high β-xylosidase. Surprisingly, we found that it produced high levels (5.36 IU mL^−1^) of β-xylosidase at 27 °C at 120 h and the production declined at 28 °C indicating that the production is sensitive to temperature at which the organism was grown (Table 1). Fermented broth containing 5.36 IU mL^−1^ was used for further purification. Native polyacrylamide gel electrophoresis in combination with zymogram staining of β-xylosidase present in the crude broth indicated that *P. hubeiensis* produced only one species of β-xylosidase (Fig. 1b). The extracellular PhXyl was purified to homogeneity from the cell free supernatant and the results are given in Table 2. Almost all β-xylosidase was adsorbed to QAE Sephadex A50 column followed by elution with 0.3 M NaCl which resulted in 5.7-fold purification with 68 % yield. Further purification with size exclusion chromatography using Sephacryl-200 gave the purified PhXyl with 53.12 % yield and specific activity of 143.12 IU mg^−1^. The mass spectrometric analysis revealed that the best match in the search of NCBInr database was with *P. hubeiensis* SY62 (gi|808364558 glycoside hydrolase). Further MASCOT searching of an in-house customised SWISS-PROT database revealed no entries that are similar to β-purified PhXyl (Additional file 1).

**Table 1:** Effect of temperature on PhXyl production

Time (h)	Temperature (°C)							
	25		27		28		30	
	pH	Xylosidase (IU/mL)	pH	Xylosidase (IU/mL)	pH	Xylosidase (IU/mL)	pH	Xylosidase (IU/mL)
24	5.18	0.11 ± 0.002	5.19	0.089 ± 0.01	5.03	0.06 ± 0.01	5.0	0.05 ± 0.002
48	6.0	0.32 ± 0.03	5.84	1.07 ± 0.03	5.66	0.09 ± 0.02	5.40	0.146 ± 0.01
72	7.07	1.91 ± 0.13	6.17	2.21 ± 0.3	6.71	1.90 ± 0.23	5.77	0.44 ± 0.1
96	7.51	2.21 ± 0.29	6.42	4.01 ± 0.5	6.75	2.63 ± 0.12	6.13	0.58 ± 0.05
120	7.80	2.33 ± 0.3	6.79	5.36 ± 0.7	6.83	2.88 ± 0.28	6.44	0.87 ± 0.03

Enzyme production was carried out at different temperatures 25, 27, 28 and 30 °C. Samples were harvested at definite interval of time and enzyme activity was calculated. The mean values and standard deviations are from three independent experiments

**Table 2:** Purification of the PhXyl

Purification steps	Total activity^a^ (IU)	Total protein (mg)	Specific activity (IU/mg)	Recovery (%)	Fold purification
Culture filtrate	1155	67.14	17.21	100.00	1
Ammonium sulphate precipitation	1099	17.86	61.42	95.20	3.56
QAE Sephadex A50 Chromatography	752	7.66	98.17	68.42	5.70
Gel filtration Chromatography (Sephacryl-200)	399.46	2.79	143.17	53.12	8.3

The values show the average of three independent experiments

^a^β-xylosidase activity was assayed using *p*NPX

### Characterization of purified PhXyl

The purity of the enzyme was confirmed by SDS-PAGE (Fig. 1a) which revealed that the molecular mass of the purified enzyme is 110 kDa. The molecular mass of native enzyme determined by MALDI-TOF showed that the purified enzyme has the molecular mass of 112.3 kDa confirming that it is a monomer. It is a glycoprotein with 23 % glycosylation.

**Fig. 2:**
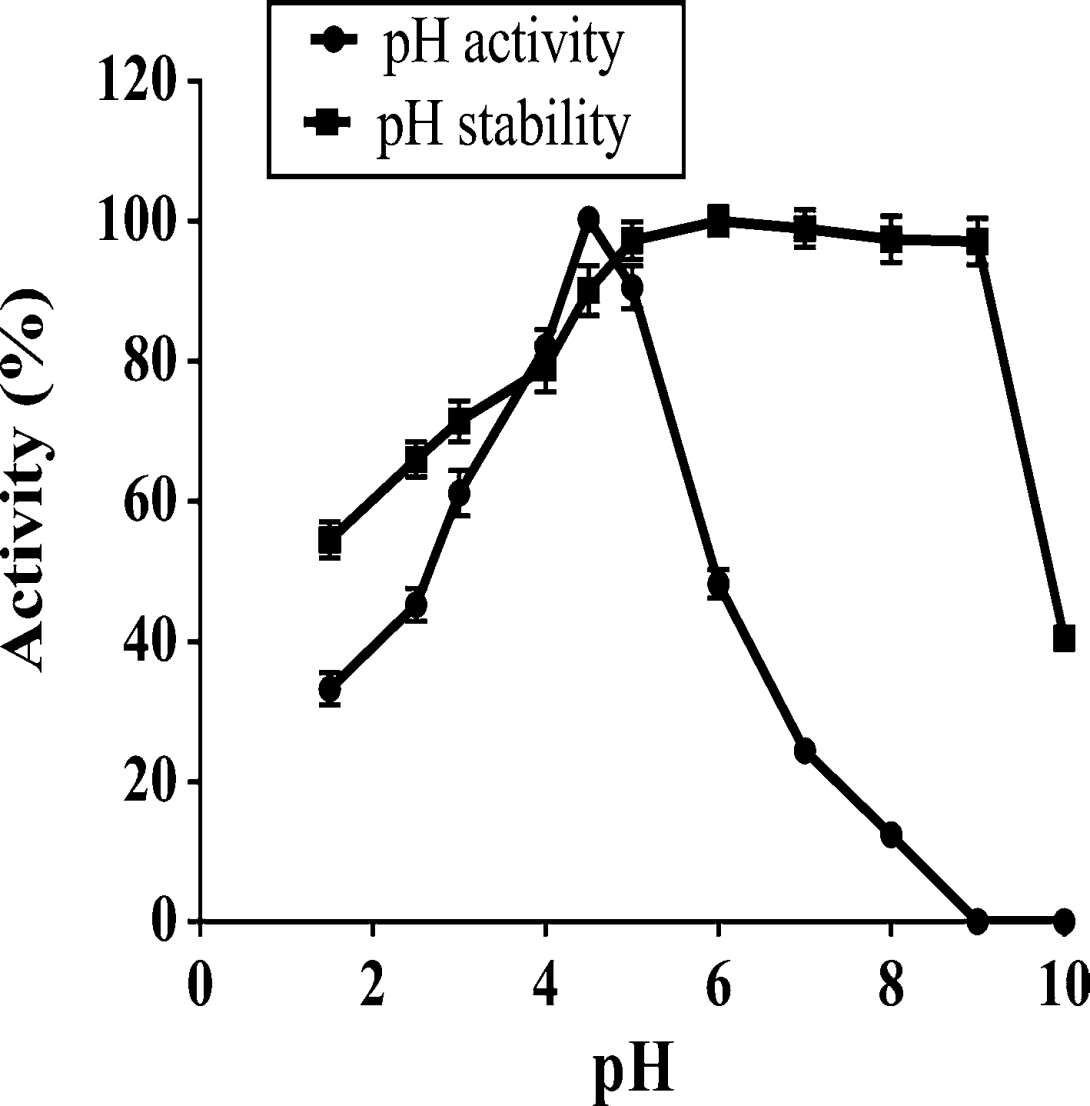
Influence of pH on PhXyl activity and stability

**Fig. 3:**
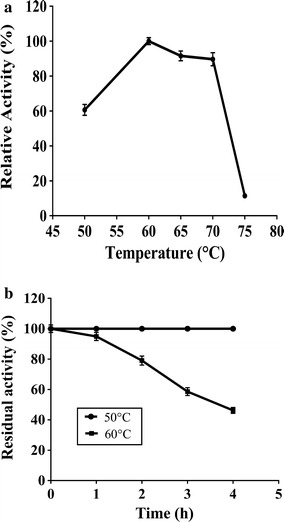
**a** Effect of temperature on the PhXyl activity, **b** effect of temperature on PhXyl stability

The purified enzyme was active at pH 4.5 and stable in a wide pH range (3.0–9.0) as it retained 75 and 100 % activity at pH 3.0 and 9.0 respectively after incubation for 24 h (Fig. 2). The enzyme exhibited broad temperature optima (55–70 °C) (Fig. 3a) and 100 and 50 % stability at 50 and 60 °C respectively (Fig. 3b). No metal ions including heavy metals such as Hg^2+^, Cu^2+^ and Ag^+^ inhibited the enzyme activity. EDTA had no influence on enzyme activity indicating no requirement of metal ions (Table 3). The kinetic parameters such as *K*_m_, *V*_max_, *K*_cat_ and *K*_cat_/*K*_m_ were found to be 0.537 mM, 314 µmol min^−1^ mg^−1^, 588.91 s^−1^ and 1096.6 mM s^−1^ respectively. The substrate specificity studies demonstrated that the purified enzyme showed highest activity towards *p*NP-β-xylopyranoside with no activity with *p*NP-β-glucopyranoside and *p*NP-α-l-arabinopyranoside.

**Table 3:** Effect of metal ions on PhXyl activity

Metal ion	Relative activity (%) in presence of metal ions^a^		
	0.1 mM	1 mM	10 mM
Control	100	100	100
HgCl_2_	86.23 ± 2.3	86.59 ± 2.0	84.70 ± 3.2
NiCl_2_	89.67 ± 3.0	98.90 ± 2.9	93.31 ± 3.6
MgCl_2_	92.20 ± 3.2	94.45 ± 2.8	88.47 ± 2.9
MnCl_2_	90.82 ± 3.0	90.82 ± 3.0	89.52 ± 3.5
CaCl_2_	89.90 ± 4.1	90.82 ± 2.78	84.91 ± 2.6
ZnCl_2_	92.88 ± 2.8	92.37 ± 3.1	91.75 ± 4.1
FeSo_4_	103.72 ± 4.3	121.72 ± 4.8	83.95 ± 3.4
FeCl_3_	110.32 ± 3.8	101.07 ± 4.1	98.61 ± 4.2
CuSo_4_	90.36 ± 2.5	90.35 ± 2.9	89.26 ± 2.2
PbCl_2_	89.22 ± 3.7	100.00 ± 3.5	96.11 ± 3.0
CoSo_4_	90.59 ± 2.9	96.14 ± 3.0	74.96 ± 2.5
AgNo_3_	98.85 ± 2.0	96.88 ± 3.7	93.31 ± 4.5
EDTA	100.00 ± 4.1	82.43 ± 2.8	73.09 ± 2.4

^a^The values show the average and standard deviation from three independent experiments

### Effect of xylose and ethanol on xylosidase

**Fig. 4:**
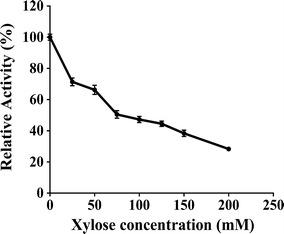
Effect of xylose on PhXyl activity. Enzyme activity was determined in presence of xylose concentration (20–200 mM) under standard assay condition

**Fig. 5:**
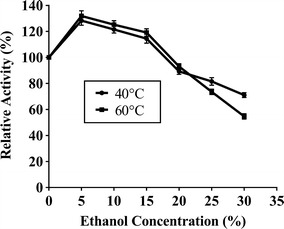
Effect of ethanol on PhXyl activity. Enzyme activity was determined in presence of various concentrations of ethanol (5–30 %) under standard assay condition

The effect of xyose on PhXyl activity was studied and the results are given in Fig. 4. Enzyme exhibited 50 % activity in presence of 75 mM of xylose. To determine type of inhibition, kinetic constants were determined and it was found that the *K*_m_ was altered while *V*_max_ remained unchanged. This suggested that xylose showed competitive inhibition (Table 4). Ethanol had no effect on PhXyl activity even at 20 % ethanol concentration. On the contrary, ethanol at 5, 10 and 15 % concentration enhanced the enzyme activity. However, the enzyme activity declined to 54 % when the assay was carried out in presence of 30 % ethanol concentration (Fig. 5).

**Table 4:** Kinetic analysis of β-xylosidase. in presence of xylose

Xylose (mM)	*K*_m_ (mM)	*V*_max_ (µmole/min/mg)
0	0.537	314.5
10	0.748	314.5
15	1.83	314.5
20	2.11	314.5

β-xylosidase activity was assayed in presence of xylose using *p*NPX at concentration from 0.23 to 5.52 mM. The values show the average of three independent experiments

### Chemical modification and substrate protection studies

The presence of amino acid functional groups required for the activity of PhXyl was determined by chemical modification studies using chemical reagents with restricted amino acid specificity. The enzyme was not inhibited by DEPC, PMSF, phenyl-glyoxal, NEM, iodoacetate, citraconic anhydride, trinotrobenzene sulphonic acid, suggesting the non-involvement of histidine, serine, arginine, cysteine, lysine residues in catalytic site (Table 5). Strong inhibition of β-xylosidase by EDAC, NBS and N-Acetylimidazole indicated the involvement of carboxyl, tryptophan and tyrosine residues for its catalytic activity.

**Table 5:** Effect of group specific modifying agents on PhXyl activity

Modifying agent	Concentration (mM)	Possible amino acid modification	Buffer systems	Residual activity (%)^a^
EDAC	200	Asx/Glx	MES/HEPES, 75:25, 50 mM pH 6	69
DEPC	5	His	Sodium phosphate 50 mM, pH 6	100
NBS	0.1	Try	Sodium acetate 50 mM, pH 4.5	00
NEM	50	Cys	Sodium phosphate 50 mM, pH 7.5	100
Iodoacetate	5	Cys	Sodium phosphate 50 mM, pH 8	100
NAI	50	Tyr	Sodium borate 50 mM, pH 7.6	62
PMSF	5	Ser	Sodium phosphate 50 mM, pH 7.5	100
Phenylglyoxal	5	Arg	Sodium bicarbonate 50 mM, pH 8.5	100
Citraconic anhydride	5	Lys	Sodium bicarbonate 50 mM, pH 8.4	100
Trinitrobenzene sulfonic acid	5	Lys	Sodium bicarbonate (4 %) 100 mM, pH 8.4	100

^a^The mean values show the average of three independent experiments

In view of these observations, the role of above-mentioned amino acid residues for catalysis was further investigated and the results are given in Table 6. The EDAC mediated inactivation was not prevented by incubating the PhXyl with excess of substrate prior to modification suggesting the involvement of carboxyl residues in catalytic activity. The NBS mediated modification of purified enzyme resulted in total loss of activity. This NBS mediated inactivation was partially prevented by pre-incubating the enzyme with excess of substrate, *p*NPX prior to modification reaction suggesting that tryptophan had a role in substrate binding. N-Acetyl imidazole at 100 mM concentration inactivated (52 %) the PhXyl. This inactivation was not reversed by pre-incubation of enzyme with excess amount of *p*NPX indicating no role of tyrosine in substrate binding site of xylosidase.

**Table 6:** Substrate protection studies

Amino acid	Reaction system	Residual activity (%)
Carboxylic acid	Buffer + enzyme	100.00
	Buffer + enzyme +200 mM EDAC	69.93
	Buffer + enzyme + 1.84 mM pNPX	75.50
	Buffer + enzyme + 3.68 mM pNPX	76.72
Tyrosine	Buffer + enzyme	100.00
	Buffer + enzyme +100 mM NAI	52.99
	Buffer + enzyme + 1.84 mM pNPX + 100 mM NAI	61.05
	Buffer + enzyme + 3.68 mM pNPX + 100 mM NAI	61.75
Tryptophan	Buffer + enzyme	100.00
	Buffer + enzyme +100 µM NBS	0.79
	Buffer + enzyme + 1.84 mM pNPX + 100 µM NBS	43.29
	Buffer + enzyme + 3.68 mM pNPX + 100 µM NBS	67.07

The PhXyl was pre-incubated with excess of *p*NPX in the respective buffers of modifying reagents. After 10 min of incubation at room temperature, the suitable concentration of modifying reagents was added and incubated for further 30 min. The aliquots were removed for the determination of residual enzyme activity under standard assay conditions. The values are the average of three independent experiments with 3-5 % standard deviation

## Discussion

*Pseudozyma hubeiensis* was first isolated in our laboratory in 1990 followed by its identification by NCYC in 2007 using 26 rDNA D1/D2 sequencing and standard taxonomic tests. This strain remained unexplored in relation to production of hydrolytic enzymes and first two papers were published from our laboratory on cellulase-free xylanase production by this yeast strain named as unidentified yeast (Bastawde et al. 1994; Gokhale et al. 1998). Such cellulase free xylanases have potential applications in pulp and paper industry where complete removal of xylan is essential to improve bio-bleaching process without the use of chlorine. Since *P. hubeiensis* was found to produce complete xylanolytic enzymes, we concentrated our efforts to purify and characterize the xylanases. The two xylanases were purified to homogeneity which produced XOS with degree of polymerization (DP) 3–7 without formation of xylose and xylobiose. These XOS produced by enzymatic hydrolysis of xylan act as useful bioactive ingredient of food and health products. These XOS are moderately sweet with no hazardous property and hence can be used in foods, juices and beverages. The XOS used as prebiotics in functional foods promote the growth of probiotic *Lactobacillus* and *Biofobacterium* species which inhibit the pathogenic bacteria preventing gastro-intestinal infections (Falck et al. 2013; Finegold et al. 2014). The complete utilization of biomass (both cellulose and hemicellulose) to obtain bulk chemicals (biofuels) and XOS makes these enzymes very interesting from industrial perspective.

In this context, the production of high amounts of β-xylosidase by *P. hubeiensis* is noteworthy. This is the first report on the production, purification and characterization of β-xylosidase from *P. hubeinsis* NCIM 3574. Maximum enzyme production (5.36 IU mL^−1^) was obtained at 27 °C at 120 h and the production declined significantly (0.87 IU mL^−1^) at 30 °C. The causes for this reduced enzyme production remained unknown. There was no significant difference in the growth of *P. hubeiensis* at temperatures ranging from 25 to 30 °C (data not shown). However, no growth and enzyme production was observed at 35 °C. This is highest β-xylosidase activity reported so far from the yeast strains (Lara et al. 2014; Otero et al. 2015). C*ryptococcus albidus* produced 1.0 IU mL^−1^ of β-xylosidase on xylan (Peciavora and Biely 1982). Guerfalli et al. (2013) reported that *Talaromyces thermophilus* produced 1.4 IU mL^−1^ of β-xylosidase in fed batch fermenter. EI-Gindy et al. (2015) reported that *A. niger* produced 5.5 IU mL^−1^ of β-xylosidase after 120 h of incubation under submerged fermentation.

The protocol used for β-xylosidase purification resulted in a final yield of 53.12 % recovered activity. During the process, the specific activity increased from 1.0 to 143.17 which implies a degree of purification of 143. Majority of the reports on β-xylosidase purification used the similar procedure which resulted in less recovery of purified enzyme (Saha 2001, 2003; Zanoelo et al. 2004; Chang et al. 2005; Katapodis et al. 2006). This is the highest recovery of purified β-xylosidase reported so far in the literature.

Many investigators have reported a wide range of *K*_m_ and *V*_max_ values for microbial β-xylosidases using *p*NPX as substrate. The *K*_m_ (0.537 mM) of the PhXyl is much lower than the values reported for intracellular β-xylosidase from *Aureobasidium* sp. (Hayashi et al. 2001) and similar to extracellular β-xylosidase from *Aureobasidium pullulan* (Dobberstein and Emeis 1991). The *V*_max_ value (314 µmol min^−1^ mg^−1^) of the PhXyl is significantly high compared to fungal enzymes from *Aspergillus japonicas* (Wakiyama et al. 2008), *Aspergillus ochraceus* (Michelin et al. 2012), *Fusarium proliferatum* (Saha 2003) and *Talaromyces amestolkiae* (Nieto-Dominquez et al. 2015). However, the *V*_max_ of β-xylosidase of *Aureobasium* sp. (Hayashi et al. 2001) is three times higher than the value obtained for PhXyl. The *K*_cat_/*K*_m_ value of the present enzyme was found to be significant indicating its superiority in catalytic efficiency.

Most of the fungal β-xylosidases are active at pH values from 4.0 to 6.0 (29). The PhXyl showed the optimum pH of 4.5 with significant activity (60 %) even at pH 3.0 and was stable in a wide pH range (3.0–9.0). The β-xylosidase of *Aureobasidium* sp. (Iembo et al. 2002) and *Penicillium sclerotiorum* (Knob and Carmona 2009) exhibited acidic pH optima of 3.0 and 2.5 respectively. Recently a novel pH stable β-xylosidase from *Talaromyces amestolkiae* was reported to display maximum activity at pH 3.0 and high stability between the pH 2.2 and 9.0 (Nieto-Dominquez et al. 2015). The enzymatic activity of the PhXyl did not vary much with the temperature around 55–70 °C with an optimum of 60 °C. It was found to be stable at 50 °C for 4 h and the retained 50 % of its original activity at 60 °C after 4 h indicating better thermostability than the enzymes from other yeast strains such as *Candida utilis* (Yanai and Sato 2001), *Pichia stipites* (Basaran and Ozcan 2008) and *Pichia membranifaciens* (Romero et al. 2012). The crude β-xylosidase of *Aureobasidium* sp. retained 75 % of its activity at 65 °C. The PhXyl also showed optimum activity and stability at high temperature indicating that this enzyme has industrially important characteristics.

Heavy metals like Hg^2+^, Ag^+^, and especially Cu^2+^ commonly inactivate the enzymes including β-xylosidases (Saha 2003; Andrade et al. 2004). The absence of inhibition of PhXyl by these metals even at 10 mM concentration was really surprising. The Cu^2+^ present in the ash content of lignocellulosic biomass reduced the yield of bioethanol production due to the cellulase inhibition caused by this metal ion (Bin and Hongzhang 2010). This property of the PhXyl is very important in considering biomass hydrolysis which contains these heavy metals.

The effect of xylose and ethanol on enzyme activity was determined and it was found that PhXyl retained 47 % of its enzyme activity in presence of 100 mM xylose concentration and the inhibition was competitive. Majority of xylosidases are inhibited at very low concentrations (2–10 mM) of xylose (Herrmann et al. 1997; Saha 2001, 2003; Zanoelo et al. 2004). Bhalla et al. (2014) reported highly thermostable β-xylosidase from *Geobacillus* WSUCF1 which is xylose resistant retaining its 50 % of activity in presence of 300 mM xylose concentration. Ethanol even at 20 % concentration did not show inhibitory effect on PhXyl activity indicating that the enzyme is ethanol tolerant. Moreover, ethanol at 5-15 % concentration was found to be the activator for enzyme activity. The enhancement in β-xylosidase activity by ethanol was reported in case of *Pichia membranifaciens* (Romero et al. 2012). The low inhibition by xylose and ethanol proved that this enzyme is a potential candidate to be used in biotechnological processes which include xylose production from xylan and ethanol production from xylose. Although many fungal enzymes have been extensively studied for xylan degradation, very few yeasts have been reported that show the ability to degrade xylan for the purpose of bioethanol production.

Chemical modification studies revealed the presence of carboxyl group containing amino acids and tryptophan in the active site of hydrolases. PhXyl contained carboxyl groups (Asx/Glx), tryptophan and tyrosine at its active site. The presence of tyrosine at active site of PhXyl is surprising since there are no reports on the presence of tyrosine at the active site of enzymes. Both carboxyl groups and tyrosine are involved in catalytic activity of PhXyl and tryptophan is involved in substrate binding.

In conclusion, *P. hubeiensis* NCIM 3574 isolated from decaying sandal wood produces a complete xylanolytic enzyme system. Two distinct xylanases have already been purified which produce XOS that have great potential as functional foods or prebiotics. It produced high levels of ethanol tolerant β-xylosidase when grown at 27 °C in submerged fermentation. The enzyme was purified to homogeneity which was found to be heavy metal and ethanol resistant. The mass spectrometric analysis revealed that the best match (26 % sequence coverage) was with *Pseudozyma hubeiensis* SY62 (gi|808364558 glycoside hydrolase). Further MASCOT searching of an in-house customised SWISS-PROT database revealed no entries that are similar to β-purified xylosidase indicating that PhXyl appears to be new. The high catalytic performance, good stability as well as activity at acidic pH and high temperatures, high metal and ethanol tolerance qualify this enzyme for the use in the hydrolysis of lignocellulosic biomass for biofuel production when mixed with efficient multi-enzyme cocktails.

## Additional file

10.1186/s13568-016-0243-7 The mass spectrometric analysis of the purified β-xylosidase from* Pseudozyma hubeiensis* NCIM 3574.

## Abbreviations

PhXyl: *Pseudozyma hubeiensis* xylosidase
SDS-PAGE: sodium dodecyl sulphate polyacrylamide gel electrophoresis
MALD-TOF: matrix-assisted laser desorption ionization time of flight
NCYC: National Collection of Yeast Cultures
NCIM: National Collection of Industrial Microorganisms
PhX: *Pseudozyma hubeiensis* xylanase
*p*NPX: *p*-nitrophenyl-β-D-xylopyranoside
*p*NPG: *p*-nitrophenyl-β-D-glucopyranoside
NEM: N-ethylmaleimide
PMSF: phenyl methylsulfonyl fluoride
DEPC: diethyl-pyrocarbonate
EDAC: 1 ethyl-3-(3 dimethyl aminopropyl) carbidiimide
TNBS: 2-4-6 trinitrobenzenesulfonic acid
NBS: 5-bromosuccinimide
NAI: N-acetylimidazole
TFA: trifluoroacetic acid

## References

[CR1] Adsul MG, Bastawde KB, Gokhale DV (2009). Biochemical characterization of two xylanases from *Pseudozyma hubeinensis* producing only xylooligosaccharides. Bioresour Technol.

[CR2] Andrade SV, Polizeli MLTM, Terenzi HF, Jorge JA (2004). Effect of carbon source on the biochemical properties of β-xylosidases produced by *Aspergillus versicolor*. Process Biochem.

[CR3] Bao L, Huang Q, Chang L, Sun Q, Zhou J, Lu H (2012). Cloning and characterization of two beta-glucosidase/xylosidase enzymes from yak rumen metagenome. Appl Biochem Biotechnol.

[CR4] Basaran P, Ozcan M (2008). Characterization of beta-xylosidase enzyme from a *Pichia stipitis* mutant. Bioresour Technol.

[CR5] Bastawde KB, Puntambekar US, Gokhale DV (1994). Optimization of cellulase free xylanase production by a novel yeast strain. J Ind Microbiol.

[CR6] Begerow D, Bauer R (2000). Phylogenic placements of ustilaginomycetous anamorphs as deduced from nuclear LSU rDNA sequences. Mycol Res.

[CR7] Bhalla A, Bischoff KM, Sani RK (2014). Highly thermostable GH39 β-xylosidase from a *Geobacillus* sp. strain WSUCF1. BMC Microbiol.

[CR8] Bin Y, Hongzhang C (2010). Effect of the ash on enzymatic hydrolysis of steam-exploded rice straw. Bioresour Technol.

[CR9] Boekhout T (1987). Systematics of anamorphs of Ustilaginales (smut fungi)—a preliminary survey. Stud Mycol.

[CR10] Chang S-C, Chou H-C, Cheng M-K, Wei D-L (2005). Purification and characterization of β-xylosidase from an isolated *Xylaria regalis* 76072314. Fung Sci.

[CR11] Chevaz R, Bull P, Eyzaguirre J (2006). The xylanolytic enzyme syatem from the genus *Penicillium*. J Biotechnol.

[CR12] Dobberstein J, Emeis CC (1991). Purification and characterization of β-xylosidase from *Aureobasidium pullulans*. Appl Microbiol Biotechnol.

[CR13] Dubois M, Gilles KA, Hamilton JK, Rebers PA, Smith F (1956). Colorimetric method for determination of sugar and related substances. Anal Chem.

[CR14] EI-Gyndi AA, Saad RR, Fawzi EM (2015). Purification of β-xylosidase from *Aspergillus tamari* using ground oats and a possible application on the fermented hydrolysate by *Pichia stipitis*. Ann Microbiol.

[CR15] Falck P, Precha-Atsawanan S, Grey C, Immerzeel P, Stålbrand H, Adlercreutz P, Karlsson EN (2013). Xylooligosaccharides from hardwood and cereal xylans produced by a thermostable xylanase as carbon sources for *Lactobacillus brevis* and *Bifidobacterium adolescentis*. J Agric Food Chem.

[CR16] Fell JW, Boekhout T, Fonseka A, Scorzetti G, Statzell-Tallman A (2000). Biodiversity and systematics of basidiomycetous yeasts as determined by large subunits rDNA D1/D2 domain sequence analysis. Int J Syst Evol Microbiol.

[CR17] Finegold SM, Li Z, Summanen PH, Downes J, Thames G, Corbett K, Dowd S, Krak M, Heber D (2014). Xylooligosaccharide increases bifidobacteria but not lactobacilli in human gut microbiota. Food Funct.

[CR18] Gokhale DV, Patil SG, Bastawde KB (1998). Potential application of yeast cellulase free xylanase in agrowaste material treatment to remove hemicellulose fraction. Bioresour Technol.

[CR19] Guerfali M, Maleej-Achouri I, Belghith H (2013). Hydrolytic potential of *Talaromyces thermophilus* β-xylosidase and its use for continuous xylose production. Food Technol Biotechnol.

[CR20] Hayashi S, Ohno T, Ito M, Yokoi H (2001). Purification and properties of the cell-associated beta-xylosidase from *Aureobasidium*. J Ind Microbiol Biotechnol.

[CR21] Herrmann MC, Vrsanska M, Jurickova M, Hirsch J, Biely P, Kubicek CP (1997). The beta-xylosidase of *Trichoderma reesei* is a multifunctional beta-d-xylan xylohydrolase. Biochem J.

[CR22] Iembo T, da Silva R, Pagnocca FC, Gomes E (2002). Production, characterization and properties of bets-glucosidase and beta-xylosidase from a strain of *Aureobasidium* sp. Appl Biochem Microbiol.

[CR23] Katapodis P, Nerinckx W, Claeyssens M, Christakopoulos P (2006). Purification and characterization of a thermostable intracellular beta-xylosidase from the thermophilic fungus *Sporotrichum thermophile*. Process Biochem.

[CR24] Knob A, Carmona EC (2009). Cell-associated acid beta-xylosidase production by *Penicillium sclerotiorum*. N Biotechnol.

[CR25] Lara CA, Santos RO, Cadete RM, Ferreira C, Marques S, Gírio F, Oliveira ES, Rosa CA, Fonseca C (2014). Identification and characterisation of xylanolytic yeasts isolated from decaying wood and sugarcane bagasse in Brazil. Antonie Van Leeuwenhoek.

[CR26] Laemmli UK (1970). Cleavage of structural proteins during assembly of head of bacteriophage-t4. Nature.

[CR27] Lowry OH, Rosebrough NJ, Farr AL, Randall RL (1951). Protein measurement with the folin phenol reagent. J Biol Chem.

[CR28] Michelin M, Polizeli MLTM, Ruzene DS, Silva DP, Vicente AA, Jorge JA, Terenzi HF, Teixeria JA (2012). Xylanase and beta-xylosidase production by *Aspergillus ochraceus*: new perspectives for the application of wheat straw autohydrolysis liquor. Appl Biochem Biotechnol.

[CR29] Nieto-Dominquez M, de Eugenio LI, Barriuso J, Prieto A, de Toro BF, Canales-Mayordomo A, Martinez MJ (2015). Novel pH-stable glycosyl hydrolase family 3 β-xylosidase from *Talaromyces amestolkiae*: an enzyme displaying regioselective transxylosylation. Appl Environ Microbiol.

[CR30] Otero DM, Cadaval CL, Teixeira LM, Rosa CA, Sanzo AVL, Kalil SJ (2015). Screening of yeasts capable of producing cellulase-free xylanase. Afr J Biotechnol.

[CR31] Padilla B, Gil JV, Manzanares P (2016). Past and future of non-saccharomyces yeasts: from spoilage microorganisms to biotechnological tools for improving wine aroma complexity. Front Microbiol.

[CR32] Peciavora A, Biely P (1982). Beta-Xylosidases and a nonspecific wall-bound beta-glucosidase of the yeast *Cryptococcus albidus*. Biochim Biophys Acta.

[CR33] Romero AM, Mateo JJ, Maicas S (2012). Characterization of an ethanol-tolerant 1,4-β-xylosidase produced by *Pichia membranifaciens*. Letts Appl Microbiol.

[CR34] Saha BC (2001). Purification and characterization of an extracellular beta-xylosidase from a newly isolated *Fusarium verticillioides*. J Ind Microbiol Biotechnol.

[CR35] Saha BC (2003). Purification and properties of an extracellular beta-xylosidase from newly isolated *Fusarium proliferatum*. Bioresour Technol.

[CR36] Wakiyama M, Yoshihara K, Hayashi S, Ohta K (2008). Purification and properties of an extracellular β-xylosidase from *Aspergillus japonicus* and sequence analysis of the encoding gene. J Biosci Bioeng.

[CR37] Wang QM, Jia J-H, Bai F-Y (2006). *Pseudozyma hubeiensis* sp. nov. and *Pseudozyma shanxiensis* sp. nov., novel ustilaginomycetous anamorphic yeast species from plant leaves. Int J Syst and Evol Microbiol.

[CR38] Yanai T, Sato M (2001). Purification and characterization of a β-d-xylosidase from *Candida utilis* IFO 0639. Biosci Biotechnol Biochem.

[CR39] Zanoelo FF, Polizeli MLTM, Terenzi HF, Jorge JA (2004). Purification and biochemical properties of a thermostable xylose-tolerant β-xylosidase from *Scytalidium thermophilum*. J Ind Microbiol Biotechnol.

