# Supplementary material for: Purification and characterization of an extracellular β-xylosidase from Pseudozyma hubeiensis NCIM 3574 (PhXyl), an unexplored yeast
Source: AMB Express. 2016 Sep 15;6:73. doi: 10.1186/s13568-016-0243-7 (PMC5023640; doi:10.1186/s13568-016-0243-7)
Supplement: Supplementary file 1 — 10.1186/s13568-016-0243-7 The mass spectrometric analysis of the purified β-xylosidase from Pseudozyma hubeiensis NCIM 3574. [file 13568_2016_243_MOESM1_ESM.pdf]

Title: Purification and characterization of an extracellular β-xylosidase from *Pseudozyma hubeiensis* NCIM 3574 (PhXyl), an unexplored yeast

Journal Name: AMB Express

Author Names: Nutan Mhetras, Susan Liddell, Digambar Gokhale

Corresponding author: Digambar Gokhale, NCIM Resource Center, CSIR-National Chemical laboratory, Pune 411008, Maharashtra, India, dv.gokhale@ncl.res.in

The purified sample labelled “B-XYLO 2015”
(aka SL2_1B in this analysis)

processed February 2016

Summary of the data

the best match of the NCBInr database search is with

gi|808364558 glycoside hydrolase [*Pseudozyma hubeiensis* SY62]

from the tandem MS data, B-XYLO 2015 is confirmed as a glycoside hydrolase/beta-glucosidase

B-XYLO 2015 tandem MS data does not match either of the two *P. janthinellum* sequence entries (described for the last sample in 2014) in the MASCOT searches

B-XYLO 2015/1B tandem MS data searched against NCBInr database version 20151016

MASCOT NCBInr search 1B 24 feb 2016

http://psbsl03/mascot/cgi/master_results_2.pl?file=20160224%2FF005316.dat
http://psbsl03/mascot/cgi/master_results.pl?file=..%2Fdata%2F20160224%2FF005316.dat

cut off = 27
http://psbsl03/mascot/cgi/master_results.pl?file=..%2Fdata%2F20160224%2FF005316.dat&_querylist=all&REPTYPE=select&_sigthreshold=0.05&REPORT=AUTO&_server_mudpit_switch=99999999&_ignoreionsscorebelow=26&_showsubsets=0&_showpopups=TRUE&_sortunassigned=scoredown&_requireboldred=0

```
MS data file            : SL2_1Bmgf.txt
Database                : NCBInr 20151016 (73055898 sequences; 26613539447 residues)
Timestamp               : 24 Feb 2016 at 20:09:35 GMT
Enzyme                  : Trypsin
Variable modifications  : Carbamidomethyl (C),Oxidation (M)
Mass values             : Monoisotopic
Protein Mass            : Unrestricted
Peptide Mass Tolerance  : ± 1.2 Da (# 13C = 2)
Fragment Mass Tolerance: ± 0.6 Da
Max Missed Cleavages    : 1
Instrument type         : ESI-FTICR
Number of queries       : 1343
Protein hits
```

```
  : gi|808364558 glycoside hydrolase [Pseudozyma hubeiensis SY62]
    gi|557997898 glycoside hydrolase [Pseudozyma brasiliensis GHG001]
    gi|343427826 related to beta-glucosidase [Sporisorium reilianum SRZ2]
    gi|674217110 glycoside hydrolase [Pseudozyma antarctica]
    gi|758987266 hypothetical protein UMAG_06075 [Ustilago maydis 521]
    gi|388857345 related to beta-glucosidase [Ustilago hordei]
    gi|673530417 related to beta-glucosidase [Melanopsichium pennsylvanicum 4]
    gi|758972508 putative beta-glucosidase [Ustilago maydis 521]
```

gi|501753225 Probable beta-glucosidase G [Taphrina deformans PYCC 5710]

Select Summary Report

Significance threshold p< 0.05	Max. number of hits AUTO	
Standard scoring (selected) MudPIT scoring	Ions score or expect cut-off 26	Show sub-sets 0

1. gi|808364558 Mass: 94016 Score: 1279 Matches: 122(68) Sequences: 17(9) emPAI: 0.93

glycoside hydrolase [Pseudozyma hubeiensis SY62]

Query	Observed	Mr(expt)	Mr(calc)	Delta	Miss	Score	Expect	Rank	Unique	Peptide
468	515.2878	1028.5611	1028.5615	-0.0003	0	51	5.3	1		K.NLVGGSDLVR.A 466 467
485	531.8076	1061.6007	1061.6022	-0.0015	0	57	1.4	1		R.LLAQWYLR.G
489	532.2925	1062.5704	1062.5709	-0.0005	0	64	0.27	1		R.AAALGSEFVAK.G 487 488
511	544.7770	1087.5394	1087.5397	-0.0003	0	57	1.4	1		R.LDADGDELIK.L 510 512 513 514
581	600.8334	1199.6522	1199.6510	0.0012	1	56	1.5	1		R.ALDAGEVKIER.I 579 580
593	608.8381	1215.6617	1215.6612	0.0005	1	47	13	1	U	R.GFDKVQGLQPK.G 594 595
635	632.8382	1263.6618	1263.6612	0.0006	1	63	0.3	1	U	K.RDFTVWDVVK.Q
642	649.8025	1297.5905	1297.5907	-0.0002	0	(74)	0.027	1		R.AGSGALMCVYNR.I 641 643
652	657.8002	1313.5859	1313.5856	0.0003	0	82	0.0038	1		R.AGSGALMCVYNR.I 653 654
667	671.8177	1341.6209	1341.6201	0.0008	0	81	0.0054	1		R.GQNEGYPTVSYK.D 668 669 670 671 672 673
719	728.8461	1455.6777	1455.6783	-0.0005	0	79	0.0071	1		K.HFLAYEQETYR.Q 716 717 718 720
723	739.8899	1477.7652	1477.7664	-0.0011	0	76	0.014	1		K.EIGEEAVTLIYNK.R 722 724

732	765.4309	1528.8472	1528.8474	-0.0002	0	82	0.0036	1		K.GVNVHLGPVTGGPLGR.S 731 733 734
757	817.9404	1633.8663	1633.8675	-0.0012	1	106	1.3e-005	1		K.EIGEEAVTLIYNKR.S 758 759 760 761
762	545.6295	1633.8667	1633.8675	-0.0008	1	(38)	93	1		K.EIGEEAVTLIYNKR.S 763 764
781	905.9320	1809.8495	1809.8509	-0.0014	0	101	3.9e-005	1		R.FGIPELCFQDGPAGFR.A 780 782 783 785 786 787 788 789 790 791 792 793 794 795 796 798 799 801
864	1056.0288	2110.0431	2110.0484	-0.0053	0	103	2.1e-005	1		R.ASDFVTVFPAGVTTAATWNR.D 862 863 865 866
868	704.3561	2110.0464	2110.0484	-0.0020	0	(91)	0.00035	1		R.ASDFVTVFPAGVTTAATWNR.D 867 869
978	832.0576	2493.1511	2493.1918	-0.0407	0	(48)	6.2	1	U	K.ENHGAVLDNLNDVGDAQNQMLVK.Q 980
981	1248.0962	2494.1779	2493.1918	0.9861	0	(53)	1.8	1	U	K.ENHGAVLDNLNDVGDAQNQMLVK.Q 982 983
989	837.7314	2510.1725	2509.1867	0.9858	0	55	1.1	1	U	K.ENHGAVLDNLNDVGDAQNQMLVK.Q 992 994
993	1256.0939	2510.1732	2509.1867	0.9865	0	(37)	80	1	U	K.ENHGAVLDNLNDVGDAQNQMLVK.Q 991
1144	1158.5430	3472.6070	3472.6212	-0.0141	0	124	1e-007	1		R.NWEGFGPDPYLHGEAAYYTVSGTQSAGVISTAK.H 1142 1143 1145 1146 1147 1149 1152 1153 1154 1155 1156 1157 1158 1159 1160 1161 1162 1163 1164 1165 1166 1167 1168 1169 1170 1171 1172 1173 1174
1150	869.1610	3472.6148	3472.6212	-0.0063	0	(108)	4e-006	1		R.NWEGFGPDPYLHGEAAYYTVSGTQSAGVISTAK.H 1148 1151

Mascot Search Results

Protein View

Match to: **gi|808364558** Score: **1279**
glycoside hydrolase [Pseudozyma hubeiensis SY62]
Found in search of SL2_1Bmgf.txt
Nominal mass (M_r): **94016**; Calculated pI value: **5.62**
NCBI BLAST search of gi|808364558 against nr
Unformatted sequence string for pasting into other applications
Taxonomy: Pseudozyma hubeiensis SY62
Links to retrieve other entries containing this sequence from NCBI Entrez:
gi|501305008 from Pseudozyma hubeiensis SY62
Variable modifications: Carbamidomethyl (C),Oxidation (M)
Cleavage by Trypsin: cuts C-term side of KR unless next residue is P

Sequence Coverage: **26%**
Matched peptides shown in **Bold Red**

1 MKAAVKSLLV LSIYAANVLS AQAASLEHLN LFLNARQADT SNQTDASFPK
51 WNISSSDNSA LATAPLAGTF YRGGPEFSDI NSPFHPVTGN GGWEWAVEKA
101 RGIVDQLTLA EKVNLTAGIT GGRCEGTLGR VDR**FGIPELC** **FQDGPAGFRA**
151 **SDFVTVFPAG** **VTTAATWNR**D LIYKR**AAALG** **SEFVAKGVNV** **HLGPVTGGPL**
201 **GR**SPFQGR**NW** **EGFGPDPYLH** **GEAAYYTVSG** **TQSAGVISTA** **KHFLAYEQET**
251 **YR**QLYAASDP YTLNPNNNTY LTYSSNVDDR TMHELYLWPF MNAVR**AGSGA**
301 **LMCVYNR**INS TQGCENSAVL NTILKDELDF QGFVVTDWSA AFNTSNTYNG
351 GSDVIMPGGM TTGGYK**NLVG** **GSDLVRALDA** **GEVKIER**IND GITR**LLAQWY**
401 **LRGQNEGYPT** **VSYK**DGYQNT IFNGSVVNEH RNVQGDHWKI VK**EIGEEAVT**
451 **LIYNKR**SNEA GPQGSTEFGL GLPLNKKARV GVFGSDAGSN PYGINSCQSW
501 IGLGSLLCPA NATSNGTQAI GWGSGAGFFP YLIDPLAGIS QVAK**ENHGAV**
551 **LDNLNDVGDA** **QNQMLVK**QAA SLTDAALVFV QARSGENSDR QSLR**LDADGD**
601 **ELIK**LVASTN NNTIVVMHTV GPVLMGDWFH HPNITALVLP HLPGQESGNS
651 LASVLYGDVN PSGKMPYSIL ADKDADHYPK IVGTPASDPQ VDFTEGLYID
701 YRAWDKMGLT PLIPFGHGIS YTNYSYSNLQ IQKSAENCYA PSAFKSNKDK
751 QPGGSGSLFQ YLVEVTASVQ NVGGMQGDEV AQLYVGYPEA ANAPIRQLR**G**
801 **FDKVQGLQPK** GEAKTATFKL TK**RDFTVWDV** **VK**QKFEVVDG EYKIWVGKSS
851 RVKDLTLKGS VTMKNGQVVG MSS

2. gi|557997898 **Mass:** 94180 **Score:** 1047 **Matches:** 86(36) **Sequences:** 15(9) **emPAI:** 0.59

glycoside hydrolase [Pseudozyma brasiliensis GHG001]

Query	Observed	Mr(expt)	Mr(calc)	Delta	Miss	Score	Expect	Rank	Unique	Peptide
225	602.3668	601.3595	601.3588	0.0007	0	22	5.2e+003	1		K.IWVGK.S
485	531.8076	1061.6007	1061.6022	-0.0015	0	57	1.4	1		R.LLAQWYLR.G
511	544.7770	1087.5394	1087.5397	-0.0003	0	57	1.4	1		R.LDADGDELIK.L 510 512 513 514
**574**	**594.8270**	**1187.6394**	**1187.6398**	**-0.0004**	**0**	**47**	**15**	**3**	**U**	**R.GVVSQLTLDEK.V** 572
581	600.8334	1199.6522	1198.6669	0.9852	1	34	2.9e+002	9		R.ALNAGEVKIER.I
642	649.8025	1297.5905	1297.5907	-0.0002	0	(74)	0.027	1		R.AGSGALMCVYNR.L 641 643
652	657.8002	1313.5859	1313.5856	0.0003	0	82	0.0038	1		R.AGSGALMCVYNR.L 653 654
667	671.8177	1341.6209	1341.6201	0.0008	0	81	0.0054	1		R.GQNEGYPTVSYK.D 668 669 670 671 672 673
719	728.8461	1455.6777	1455.6783	-0.0005	0	79	0.0071	1		K.HFLAYEQETYR.Q 716 717 718 720
723	739.8899	1477.7652	1477.7664	-0.0011	0	76	0.014	1		K.EIGEEAVTLIYNK.R 722 724
730	510.6227	1528.8463	1528.8474	-0.0012	0	(26)	1.5e+003	10		K.GVNVHLGPVTGGPLGR.S
732	765.4309	1528.8472	1528.8474	-0.0002	0	82	0.0036	1		K.GVNVHLGPVTGGPLGR.S 731 733 734
**740**	**527.9458**	**1580.8155**	**1580.8158**	**-0.0003**	**1**	**(36)**	**1.4e+002**	**1**		**R.HSLRLDADGDELIK.L**
**741**	**791.4160**	**1580.8175**	**1580.8158**	**0.0017**	**1**	**52**	**3.9**	**1**		**R.HSLRLDADGDELIK.L**
757	817.9404	1633.8663	1633.8675	-0.0012	1	106	1.3e-005	1		K.EIGEEAVTLIYNKR.S 758 759 760 761
762	545.6295	1633.8667	1633.8675	-0.0008	1	(38)	93	1		K.EIGEEAVTLIYNKR.S 763 764
781	905.9320	1809.8495	1809.8509	-0.0014	0	101	3.9e-005	1		R.FGIPELCFQDGPAGFR.A 780 782 783 785 786 787 788 789 790 791 792 793 794 795 796 798 799 801
**819**	**945.4875**	**1888.9604**	**1888.9658**	**-0.0054**	**0**	**74**	**0.021**	**1**		**R.TLHELYLWPFMNAVR.A** 814 815 816 817 818 823
**822**	**630.6614**	**1888.9624**	**1888.9658**	**-0.0034**	**0**	**(51)**	**4**	**1**		**R.TLHELYLWPFMNAVR.A** 820 821
**830**	**953.4887**	**1904.9629**	**1904.9607**	**0.0022**	**0**	**(57)**	**0.94**	**1**		**R.TLHELYLWPFMNAVR.A** 828 829
864	1056.0288	2110.0431	2110.0484	-0.0053	0	103	2.1e-005	1		R.ASDFVTVFPAGVTTAATWNR.D 862 863 865 866
868	704.3561	2110.0464	2110.0484	-0.0020	0	(91)	0.00035	1		R.ASDFVTVFPAGVTTAATWNR.D 867 869

3. gi|343427826 **Mass:** 93869 **Score:** 1016 **Matches:** 113(65) **Sequences:** 14(8) **emPAI:** 0.59

related to beta-glucosidase [Sporisorium reilianum SRZ2]

Query	Observed	Mr(expt)	Mr(calc)	Delta	Miss	Score	Expect	Rank	Unique	Peptide
225	602.3668	601.3595	601.3588	0.0007	0	22	5.2e+003	1		K.IWVGK.S
468	515.2878	1028.5611	1028.5615	-0.0003	0	51	5.3	1		K.NLVGGSDLVR.A 466 467
511	544.7770	1087.5394	1087.5397	-0.0003	0	57	1.4	1		R.LDADGDELIK.L 510 512 513 514
581	600.8334	1199.6522	1198.6669	0.9852	1	34	2.9e+002	9		R.ALNAGEVKIER.I
667	671.8177	1341.6209	1341.6565	-0.0356	1	63	0.31	2	U	R.GQDKGYPTVSYK.D 668 669 670 671 672 673
719	728.8461	1455.6777	1455.6783	-0.0005	0	79	0.0071	1		K.HFLAYEQETYR.Q 716 717 718 720
723	739.8899	1477.7652	1477.7664	-0.0011	0	76	0.014	1		K.EIGEEAVTLIYNK.R 722 724
730	510.6227	1528.8463	1528.8474	-0.0012	0	(26)	1.5e+003	10		K.GVNVHLGPVTGGPLGR.S
732	765.4309	1528.8472	1528.8474	-0.0002	0	82	0.0036	1		K.GVNVHLGPVTGGPLGR.S 731 733 734
740	527.9458	1580.8155	1580.8158	-0.0003	1	(36)	1.4e+002	1		R.HSLRLDADGDELIK.L
741	791.4160	1580.8175	1580.8158	0.0017	1	52	3.9	1		R.HSLRLDADGDELIK.L
757	817.9404	1633.8663	1633.8675	-0.0012	1	106	1.3e-005	1		K.EIGEEAVTLIYNKR.S 758 759 760 761
762	545.6295	1633.8667	1633.8675	-0.0008	1	(38)	93	1		K.EIGEEAVTLIYNKR.S 763 764
781	905.9320	1809.8495	1809.8509	-0.0014	0	101	3.9e-005	1		R.FGIPELCFQDGPAGFR.A 780 782 783 785 786 787 788 789 790 791 792 793 794 795 796 798 799 801
819	945.4875	1888.9604	1888.9658	-0.0054	0	74	0.021	1		R.TLHELYLWPFMNAVR.A 814 815 816 817 818 823
822	630.6614	1888.9624	1888.9658	-0.0034	0	(51)	4	1		R.TLHELYLWPFMNAVR.A 820 821
830	953.4887	1904.9629	1904.9607	0.0022	0	(57)	0.94	1		R.TLHELYLWPFMNAVR.A 828 829
864	1056.0288	2110.0431	2110.0484	-0.0053	0	103	2.1e-005	1		R.ASDFVTVFPAGVTTAATWNR.D 862 863 865 866
868	704.3561	2110.0464	2110.0484	-0.0020	0	(91)	0.00035	1		R.ASDFVTVFPAGVTTAATWNR.D 867 869
1144	1158.5430	3472.6070	3472.6212	-0.0141	0	124	1e-007	1		R.NWEGFGPDPYLHGEAAYYTVSGTQSAGVISTAK.H 1142 1143 1145 1146 1147 1149 1152 1153 1154 1155 1156 1157 1158 1159 1160 1161 1162 1163 11[illegible]
1150	869.1610	3472.6148	3472.6212	-0.0063	0	(108)	4e-006	1		R.NWEGFGPDPYLHGEAAYYTVSGTQSAGVISTAK.H 1148 1151

4. gi|443898943 **Mass:** 94076 **Score:** 1011 **Matches:** 99(41) **Sequences:** 15(7) **emPAI:** 0.59

hypothetical protein PANT_20d00032 [Pseudozyma antarctica T-34]

Query	Observed	Mr(expt)	Mr(calc)	Delta	Miss	Score	Expect	Rank	Unique	Peptide
225	602.3668	601.3595	601.3588	0.0007	0	22	5.2e+003	1		K.IWVGK.S
454	492.2384	982.4622	982.4621	0.0001	0	22	4.1e+003	5		R.NVQGDHWK.I 455
468	515.2878	1028.5611	1028.5615	-0.0003	0	51	5.3	1		K.NLVGGSDLVR.A 466 467
511	544.7770	1087.5394	1087.5397	-0.0003	0	57	1.4	1		R.LDADGDELIK.L 510 512 513 514
581	600.8334	1199.6522	1199.6510	0.0012	1	56	1.5	1		R.ALDAGEVKIER.I 579 580
719	728.8461	1455.6777	1455.6783	-0.0005	0	79	0.0071	1		K.HFLAYEQETYR.Q 716 717 718 720
723	739.8899	1477.7652	1477.7664	-0.0011	0	63	0.31	2	U	K.EVAEEAVTLIYNK.A 722 724
730	510.6227	1528.8463	1528.8474	-0.0012	0	(26)	1.5e+003	10		K.GVNVHLGPVTGGPLGR.G
732	765.4309	1528.8472	1528.8474	-0.0002	0	82	0.0036	1		K.GVNVHLGPVTGGPLGR.G 731 733 734
740	527.9458	1580.8155	1580.8158	-0.0003	1	(36)	1.4e+002	1		R.HSLRLDADGDELIK.L
741	791.4160	1580.8175	1580.8158	0.0017	1	52	3.9	1		R.HSLRLDADGDELIK.L
752	**808.9396**	**1615.8647**	**1615.8682**	**-0.0035**	**0**	**75**	**0.018**	**2**	**U**	**K.QAAGLADASLVFVQAR.S** 750 751 753 754
781	905.9320	1809.8495	1809.8509	-0.0014	0	101	3.9e-005	1		R.FGIPELCFQDGPAGFR.A 780 782 783 785 786 787 788 789 790 791 792 793 794 795 796 798 799 801
819	945.4875	1888.9604	1888.9658	-0.0054	0	74	0.021	1		R.TLHELYLWPFMNAVR.A 814 815 816 817 818 823
822	630.6614	1888.9624	1888.9658	-0.0034	0	(51)	4	1		R.TLHELYLWPFMNAVR.A 820 821
830	953.4887	1904.9629	1904.9607	0.0022	0	(57)	0.94	1		R.TLHELYLWPFMNAVR.A 828 829
864	1056.0288	2110.0431	2110.0484	-0.0053	0	103	2.1e-005	1		R.ASDFVTVFPAGVTTAATWNR.D 862 863 865 866
868	704.3561	2110.0464	2110.0484	-0.0020	0	(91)	0.00035	1		R.ASDFVTVFPAGVTTAATWNR.D 867 869
951	**1221.5925**	**2441.1705**	**2441.1751**	**-0.0046**	**0**	**(121)**	**3.5e-007**	**1**		**R.IVGSPASDPQVDFTEGLYIDYR.A** 949 950 952 953 961 963 969
954	**814.7310**	**2441.1713**	**2441.1751**	**-0.0038**	**0**	**134**	**1.8e-008**	**1**		**R.IVGSPASDPQVDFTEGLYIDYR.A** 955 956 957 958 959 960 962 964 965 966 967 968 970 971 972
1178	**1162.8779**	**3485.6118**	**3486.6368**	**-1.0250**	**0**	**47**	**4.5**	**1**	**U**	**R.NWEGFGPDPYLHGEAAYYTVSGTQSAGVITTAK.H**

Proteins matching the same set of peptides:

gi|573027117 **Mass:** 94018 **Score:** 1007 **Matches:** 99(41) **Sequences:** 15(7)

hypothetical protein PaG_05571 [Pseudozyma aphidis DSM 70725]

5. gi|758987266 **Mass:** 94463 **Score:** 673 **Matches:** 74(48) **Sequences:** 9(6) **emPAI:** 0.36

hypothetical protein UMAG_06075 [Ustilago maydis 521]

Query	Observed	Mr(expt)	Mr(calc)	Delta	Miss	Score	Expect	Rank	Unique	Peptide
225	602.3668	601.3595	601.3588	0.0007	0	22	5.2e+003	1		K.IWVGK.S
468	515.2878	1028.5611	1028.5615	-0.0003	0	51	5.3	1		K.NLVGGSDLVR.A 466 467
485	531.8076	1061.6007	1061.6022	-0.0015	0	57	1.4	1		R.LLAQWYLR.G
719	728.8461	1455.6777	1455.6783	-0.0005	0	79	0.0071	1		K.HFLAYEQETYR.Q 716 717 718 720
730	510.6227	1528.8463	1528.8474	-0.0012	0	(26)	1.5e+003	10		K.GVNVHLGPVTGGPLGR.S
732	765.4309	1528.8472	1528.8474	-0.0002	0	82	0.0036	1		K.GVNVHLGPVTGGPLGR.S 731 733 734
751	808.9396	1615.8647	1615.8682	-0.0035	0	117	1.2e-006	1	U	K.QAAGLSDAALVFVQAR.S 750 752 753 754
819	945.4875	1888.9604	1888.9658	-0.0054	0	74	0.021	1		R.TLHELYLWPFMNAVR.A 814 815 816 817 818 823
822	630.6614	1888.9624	1888.9658	-0.0034	0	(51)	4	1		R.TLHELYLWPFMNAVR.A 820 821
830	953.4887	1904.9629	1904.9607	0.0022	0	(57)	0.94	1		R.TLHELYLWPFMNAVR.A 828 829
862	1056.0288	2110.0431	2111.0324	-0.9893	0	71	0.034	2	U	R.ASDFVTVFPAGVTTAATWDR.D 863 864 865 866
867	704.3561	2110.0464	2111.0324	-0.9860	0	(44)	16	2	U	R.ASDFVTVFPAGVTTAATWDR.D 868 869
1144	1158.5430	3472.6070	3472.6212	-0.0141	0	124	1e-007	1		R.NWEGFGPDPYLHGEAAYYTVSGTQSAGVISTAK.H 1142 1143 1145 1146 1147 1149 1152 1153 1154 1155 1156 1157 1158 1159 1160 1161 1162 1163 11
1150	869.1610	3472.6148	3472.6212	-0.0063	0	(108)	4e-006	1		R.NWEGFGPDPYLHGEAAYYTVSGTQSAGVISTAK.H 1148 1151

6. gi|388857345 **Mass:** 90310 **Score:** 647 **Matches:** 87(34) **Sequences:** 9(6) **emPAI:** 0.32

related to beta-glucosidase [Ustilago hordei]

Query	Observed	Mr(expt)	Mr(calc)	Delta	Miss	Score	Expect	Rank	Unique	Peptide
225	602.3668	601.3595	601.3588	0.0007	0	22	5.2e+003	1		K.IWVGK.S

454	492.2384	982.4622	982.4621	0.0001	0	22	4.1e+003	5		R.NVQGDHWK.I 455
485	531.8076	1061.6007	1061.6022	-0.0015	0	57	1.4	1		R.LLAQWYLR.G
572	594.8269	1187.6393	1187.6398	-0.0005	0	78	0.011	1	U	R.SVVGQLTLDEK.V 573 574
719	728.8461	1455.6777	1455.6783	-0.0005	0	79	0.0071	1		K.HFLAYEQETYR.Q 716 717 718 720
730	510.6227	1528.8463	1528.8474	-0.0012	0	(26)	1.5e+003	10		K.GVNVHLGPVTGGPLGR.S
732	765.4309	1528.8472	1528.8474	-0.0002	0	82	0.0036	1		K.GVNVHLGPVTGGPLGR.S 731 733 734
781	905.9320	1809.8495	1809.8509	-0.0014	0	101	3.9e-005	1		R.FGIPELCFQDGPAGFR.A 780 782 783 785 786 787 788 789 790 791 792 793 794 795 796 798 799 801
951	1221.5925	2441.1705	2441.1751	-0.0046	0	(121)	3.5e-007	1		K.IVGSPASDPQVDFTEGLYIDYR.A 949 950 952 953 961 963 969
954	814.7310	2441.1713	2441.1751	-0.0038	0	134	1.8e-008	1		K.IVGSPASDPQVDFTEGLYIDYR.A 955 956 957 958 959 960 962 964 965 966 967 968 970 971 972
1100	**967.1145**	**2898.3218**	**2897.3409**	**0.9808**	**0**	**74**	**0.013**	**1**	**U**	**R.GGPEFADLNSPFHPVTGNGGWEWAVEK.A** 1092 1093 1094 1095 1098 1099 1101 1103 1104 1105 1106 1107 1108 1109 1110 1111 1112 1113 1114 1115

---

7. gi|673530417 **Mass:** 94733 **Score:** 531 **Matches:** 55(18) **Sequences:** 8(4) **emPAI:** 0.31

related to beta-glucosidase [Melanopsichium pennsylvanicum 4]

Query	Observed	Mr(expt)	Mr(calc)	Delta	Miss	Score	Expect	Rank	Unique	Peptide
454	492.2384	982.4622	982.4621	0.0001	0	22	4.1e+003	5		R.NVQGDHWK.I 455
489	532.2925	1062.5704	1062.5709	-0.0005	0	64	0.27	1		R.AAALGSEFVAK.G 487 488
635	632.8382	1263.6618	1263.6612	0.0006	1	46	15	2	U	K.RDFSIWDVVK.Q
667	671.8177	1341.6209	1341.6201	0.0008	0	63	0.31	2	U	R.GQDQGYPTVSYK.D 668 669 670 671 672 673
719	728.8461	1455.6777	1455.6783	-0.0005	0	79	0.0071	1		K.HFLAYEQETYR.Q 716 717 718 720
730	510.6227	1528.8463	1528.8474	-0.0012	0	(26)	1.5e+003	10		K.GVNVHLGPVTGGPLGR.S
732	765.4309	1528.8472	1528.8474	-0.0002	0	82	0.0036	1		K.GVNVHLGPVTGGPLGR.S 731 733 734
781	905.9320	1809.8495	1809.8509	-0.0014	0	101	3.9e-005	1		R.FGIPELCFQDGPAGFR.A 780 782 783 785 786 787 788 789 790 791 792 793 794 795 796 798 799 801
819	945.4875	1888.9604	1888.9658	-0.0054	0	74	0.021	1		R.TLHELYLWPFMNAVR.A 814 815 816 817 818 823
822	630.6614	1888.9624	1888.9658	-0.0034	0	(51)	4	1		R.TLHELYLWPFMNAVR.A 820 821
830	953.4887	1904.9629	1904.9607	0.0022	0	(57)	0.94	1		R.TLHELYLWPFMNAVR.A 828 829

---

8. gi|758972508 **Mass:** 90660 **Score:** 96 **Matches:** 4(1) **Sequences:** 2(1) **emPAI:** 0.04

putative beta-glucosidase [Ustilago maydis 521]

Query	Observed	Mr(expt)	Mr(calc)	Delta	Miss	Score	Expect	Rank	Unique	Peptide
1088	**934.7882**	**2801.3427**	**2799.3697**	**1.9730**	**0**	**28**	**5.7e+002**	**1**	**U**	**R.ILAGWFLLGQDQGFPEPNFNFFDK.M**
1139	**1145.5507**	**3433.6303**	**3433.6103**	**0.0200**	**0**	**69**	**0.034**	**1**	**U**	**R.SWEGFGADPYLNGEGSYFSVLGAQDAGVQATLK.H** 1140 1141

---

9. gi|501753225 **Mass:** 80878 **Score:** 79 **Matches:** 5(5) **Sequences:** 1(1) **emPAI:** 0.05

Probable beta-glucosidase G [Taphrina deformans PYCC 5710]

Query	Observed	Mr(expt)	Mr(calc)	Delta	Miss	Score	Expect	Rank	Unique	Peptide
719	728.8461	1455.6777	1455.6783	-0.0005	0	79	0.0071	1	U	K.HFIAYEQETYR.W 716 717 718 720

Proteins matching the same set of peptides:

gi|761926286 **Mass:** 84115 **Score:** 79 **Matches:** 5(5) **Sequences:** 1(1)

beta-D-xylosidase/beta-D-glucosidase [Fistulina hepatica ATCC 64428]

gi|761932289 **Mass:** 85132 **Score:** 79 **Matches:** 5(5) **Sequences:** 1(1)

putative beta-glucosidase [Fistulina hepatica ATCC 64428]

---

search of customised swissprot DB 24 Feb 2016

swissprot supplemented with the 2 *Penicillium janthinellum* entries found previously on the genome site

```
jgi|Penja1|430813|fgenesh1_kg.3_#_629_#_Locus7268v1rpkm6.79
jgi|Penja1|390814|estExt_Genewise1.C_7_t10162
```

no statistically significant matches are found in this search (I have pasted in the output below)

this means that the purified sample is NOT similar to either of the two *P.j*. database entries that I obtained from the genome sequence site in October 2014

it also means that SWISSPROT does not contain any entries that are similar to the purified sample

search output of customised swissprot DB search 24 Feb 2016 *note no matches from this search*

http://psbsl03/mascot/cgi/master_results.pl?file=..%2Fdata%2F20160224%2FF005314.dat&_querylist=all&REPTYPE=peptide&_sigthreshold=0.05&REPORT=AUTO&_server_mudpit_switch=0.000000001&_ignoreionsscorebelow=0&_showsubsets=0&_showpopups=TRUE&_sortunassigned=scoredown&_requireboldred=0

MS data file : SL2_1Bmgf.txt
Database : SWISS_Pjan2 Pjan2 (550118 sequences; 196220699 residues)
Timestamp : 24 Feb 2016 at 17:17:21 GMT
Protein hits :

sp|A1S547|HSCB_SHEAM	Co-chaperone protein HscB homolog OS=Shewanella amazonensis (strain ATCC BAA-1098 / SB2B) GN=hscB PE=3 SV=1
sp|A5IHQ9|RL22_LEGPC	50S ribosomal protein L22 OS=Legionella pneumophila (strain Corby) GN=rplV PE=3 SV=1
sp|A5IJW7|RL11_THEP1	50S ribosomal protein L11 OS=Thermotoga petrophila (strain RKU-1 / ATCC BAA-488 / DSM 13995) GN=rplK PE=3 SV=1
sp|Q12122|HOSM_YEAST	Homocitrate synthase, mitochondrial OS=Saccharomyces cerevisiae (strain ATCC 204508 / S288c) GN=LYS21 PE=1 SV=1
sp|Q5ZYN8|RL22_LEGPH	50S ribosomal protein L22 OS=Legionella pneumophila subsp. pneumophila (strain Philadelphia 1 / ATCC 33152 / DSM 7513) GN=rplV PE=3 SV=1
sp|A0AF11|PTH_LISW6	Peptidyl-tRNA hydrolase OS=Listeria welshimeri serovar 6b (strain ATCC 35897 / DSM 20650 / SLCC5334) GN=pth PE=3 SV=1
sp|Q2LW77|ACSA_SYNAS	Acetyl-coenzyme A synthetase OS=Syntrophus aciditrophicus (strain SB) GN=acsA PE=3 SV=1
sp|Q5RG49|ACSF4_DANRE	Acyl-CoA synthetase family member 4 OS=Danio rerio GN=aasdh PE=3 SV=1

Mascot Score Histogram

Ions score is -10*Log(P), where P is the probability that the observed match is a random event.
Individual ions scores > 49 indicate identity or extensive homology (p<0.05).
Protein scores are derived from ions scores as a non-probabilistic basis for ranking protein hits.

Peptide Summary Report

1. sp|A1S547|HSCB_SHEAM **Mass:** 20024 **Score:** 50 **Matches:** 3(0) **Sequences:** 1(0) **emPAI:** 0.19
Co-chaperone protein HscB homolog OS=Shewanella amazonensis (strain ATCC BAA-1098 / SB2B) GN=hscB PE=3 SV=1

	Query	Observed	Mr(expt)	Mr(calc)	Delta	Miss	Score	Expect	Rank	Unique	Peptide
☑	319	**790.4200**	**789.4127**	**786.4963**	**2.9164**	**1**	**(48)**	**0.12**	**1**	**U**	**K.QVTAKLK.T**
☑	320	**790.4200**	**789.4127**	**786.4963**	**2.9164**	**1**	**48**	**0.11**	**1**	**U**	**K.QVTAKLK.T**
☑	321	**790.4200**	**789.4127**	**786.4963**	**2.9164**	**1**	**(32)**	**5**	**1**	**U**	**K.QVTAKLK.T**

2. sp|A5IHQ9|RL22_LEGPC **Mass:** 12013 **Score:** 50 **Matches:** 3(0) **Sequences:** 1(0) **emPAI:** 0.33
50S ribosomal protein L22 OS=Legionella pneumophila (strain Corby) GN=rplV PE=3 SV=1

Query	Observed	Mr(expt)	Mr(calc)	Delta	Miss	Score	Expect	Rank	Unique	Peptide
319	790.4200	789.4127	787.4803	1.9324	1	(48)	0.12	1	U	M.EVTAKLK.G
320	790.4200	789.4127	787.4803	1.9324	1	48	0.11	1	U	M.EVTAKLK.G
321	790.4200	789.4127	787.4803	1.9324	1	(32)	5	1	U	M.EVTAKLK.G

Proteins matching the same set of peptides:
sp|Q5WZK7|RL22_LEGPL **Mass:** 12013 **Score:** 50 **Matches:** 3(0) **Sequences:** 1(0)
50S ribosomal protein L22 OS=Legionella pneumophila (strain Lens) GN=rplV PE=3 SV=1
sp|Q5X854|RL22_LEGPA **Mass:** 12013 **Score:** 50 **Matches:** 3(0) **Sequences:** 1(0)
50S ribosomal protein L22 OS=Legionella pneumophila (strain Paris) GN=rplV PE=3 SV=1

3. sp|A5IJW7|RL11_THEP1 **Mass:** 15140 **Score:** 50 **Matches:** 3(0) **Sequences:** 1(0) **emPAI:** 0.26
50S ribosomal protein L11 OS=Thermotoga petrophila (strain RKU-1 / ATCC BAA-488 / DSM 13995) GN=rplK PE=3 SV=1

Query	Observed	Mr(expt)	Mr(calc)	Delta	Miss	Score	Expect	Rank	Unique	Peptide
319	790.4200	789.4127	786.4963	2.9164	1	(48)	0.12	1	U	K.KVTAQIK.L
320	790.4200	789.4127	786.4963	2.9164	1	48	0.11	1	U	K.KVTAQIK.L
321	790.4200	789.4127	786.4963	2.9164	1	(32)	5	1	U	K.KVTAQIK.L

4. sp|Q12122|HOSM_YEAST **Mass:** 48564 **Score:** 50 **Matches:** 3(0) **Sequences:** 1(0) **emPAI:** 0.08
Homocitrate synthase, mitochondrial OS=Saccharomyces cerevisiae (strain ATCC 204508 / S288c) GN=LYS21 PE=1 SV=1

Query	Observed	Mr(expt)	Mr(calc)	Delta	Miss	Score	Expect	Rank	Unique	Peptide
319	790.4200	789.4127	787.4803	1.9324	1	(48)	0.12	1	U	K.EVTAKIK.K
320	790.4200	789.4127	787.4803	1.9324	1	48	0.11	1	U	K.EVTAKIK.K
321	790.4200	789.4127	787.4803	1.9324	1	(32)	5	1	U	K.EVTAKIK.K

Proteins matching the same set of peptides:
sp|P48570|HOSC_YEAST **Mass:** 47069 **Score:** 50 **Matches:** 3(0) **Sequences:** 1(0)
Homocitrate synthase, cytosolic isozyme OS=Saccharomyces cerevisiae (strain ATCC 204508 / S288c) GN=LYS20 PE=1 SV=1

5. sp|Q5ZYN8|RL22_LEGPH **Mass:** 12144 **Score:** 37 **Matches:** 2(0) **Sequences:** 1(0) **emPAI:** 0.33
50S ribosomal protein L22 OS=Legionella pneumophila subsp. pneumophila (strain Philadelphia 1 / ATCC 33152 / DSM 7513) GN=rplV PE=3 SV=1

	Query	Observed	Mr(expt)	Mr(calc)	Delta	Miss	Score	Expect	Rank	Unique	Peptide
☑	474	**1053.0495**	**1052.0422**	**1049.5613**	**2.4810**	**1**	**(30)**	**4.4**	**1**	**U**	**-.MMEVTAKLK.G**
☑	476	**1053.5503**	**1052.5430**	**1049.5613**	**2.9817**	**1**	**37**	**1.1**	**1**	**U**	**-.MMEVTAKLK.G**

6. sp|A0AF11|PTH_LISW6 **Mass:** 20738 **Score:** 34 **Matches:** 5(0) **Sequences:** 1(0) **emPAI:** 0.18
Peptidyl-tRNA hydrolase OS=Listeria welshimeri serovar 6b (strain ATCC 35897 / DSM 20650 / SLCC5334) GN=pth PE=3 SV=1

	Query	Observed	Mr(expt)	Mr(calc)	Delta	Miss	Score	Expect	Rank	Unique	Peptide
☑	862	**1056.0288**	**2110.0431**	**2109.0089**	**1.0343**	**0**	**(17)**	**79**	**1**	**U**	**K.SADAIEDFAQVPFVEVMNK.Y**
☑	863	**1056.0288**	**2110.0431**	**2109.0089**	**1.0343**	**0**	**(23)**	**17**	**1**	**U**	**K.SADAIEDFAQVPFVEVMNK.Y**
☑	864	**1056.0288**	**2110.0431**	**2109.0089**	**1.0343**	**0**	**34**	**1.4**	**1**	**U**	**K.SADAIEDFAQVPFVEVMNK.Y**
☑	865	**1056.0288**	**2110.0431**	**2109.0089**	**1.0343**	**0**	**(17)**	**75**	**1**	**U**	**K.SADAIEDFAQVPFVEVMNK.Y**
☑	866	**1056.0288**	**2110.0431**	**2109.0089**	**1.0343**	**0**	**(23)**	**18**	**1**	**U**	**K.SADAIEDFAQVPFVEVMNK.Y**

7. sp|Q2LW77|ACSA_SYNAS **Mass:** 72283 **Score:** 33 **Matches:** 8(0) **Sequences:** 1(0) **emPAI:** 0.05
Acetyl-coenzyme A synthetase OS=Syntrophus aciditrophicus (strain SB) GN=acsA PE=3 SV=1

	Query	Observed	Mr(expt)	Mr(calc)	Delta	Miss	Score	Expect	Rank	Unique	Peptide
☑	757	**817.9404**	**1633.8663**	**1634.7610**	**-0.8947**	**1**	**(32)**	**2.5**	**1**	**U**	**K.ENADEAVGKCPFVEK.V**
☑	758	**817.9405**	**1633.8665**	**1634.7610**	**-0.8945**	**1**	**(31)**	**3.5**	**1**	**U**	**K.ENADEAVGKCPFVEK.V**

☑	759	817.9406	1633.8666	1634.7610	-0.8944	1	(33)	2.3	1	U	K.ENADEAVGKCPFVEK.V
☑	760	817.9406	1633.8666	1634.7610	-0.8944	1	33	2.2	1	U	K.ENADEAVGKCPFVEK.V
☑	761	817.9406	1633.8666	1634.7610	-0.8944	1	(28)	6.4	1	U	K.ENADEAVGKCPFVEK.V
	762	545.6295	1633.8667	1634.7610	-0.8943	1	(21)	37	2	U	K.ENADEAVGKCPFVEK.V
	763	545.6296	1633.8669	1634.7610	-0.8941	1	(11)	3.3e+002	8	U	K.ENADEAVGKCPFVEK.V
	764	545.6296	1633.8670	1634.7610	-0.8941	1	(15)	1.5e+002	3	U	K.ENADEAVGKCPFVEK.V

8. sp|Q5RG49|ACSF4_DANRE **Mass:** 125426 **Score:** 14 **Matches:** 1(0) **Sequences:** 1(0) **emPAI:** 0.03

Acyl-CoA synthetase family member 4 OS=Danio rerio GN=aasdh PE=3 SV=1

	Query	Observed	Mr(expt)	Mr(calc)	Delta	Miss	Score	Expect	Rank	Unique	Peptide
☑	651	1313.2500	1312.2427	1312.7139	-0.4712	0	14	1.5e+002	1	U	K.LLYELVHEAAR.A
